# *Theileria orientalis* Ikeda Genotype in Cattle, Virginia, USA

**DOI:** 10.3201/eid2509.190088

**Published:** 2019-09

**Authors:** Vanessa J. Oakes, Michael J. Yabsley, Diana Schwartz, Tanya LeRoith, Carolynn Bissett, Charles Broaddus, Jack L. Schlater, S. Michelle Todd, Katie M. Boes, Meghan Brookhart, Kevin K. Lahmers

**Affiliations:** Virginia–Maryland College of Veterinary Medicine, Blacksburg, Virginia, USA (V.J. Oakes, T. LeRoith, S.M. Todd, K.M. Boes, M. Brookhart, K.K. Lahmers);; University of Georgia, Athens, Georgia, USA (M.J. Yabsley);; Kansas State University, Manhattan, Kansas, USA (D. Schwartz);; Virginia Department of Agriculture and Consumer Services, Richmond, Virginia, USA (C. Bissett, C. Broaddus);; US Department of Agriculture, Ames, Iowa, USA (J.L. Schlater)

**Keywords:** Theileria orientalis, Ikeda genotype, parasites, theileriosis, cattle, infectious disease, ticks, Haemaphysalis longicornis, Ixodidae, anemia, zoonoses, tick-borne infections, vector-borne infections, Virginia, United States

## Abstract

*Theileria orientalis* Ikeda genotype is a parasite that causes a disease in cattle that results in major economic issues in Asia, New Zealand, and Australia. The parasite is transmitted by *Haemaphysalis longicornis* ticks, which have recently been reported in numerous states throughout the eastern United States. Concurrently, cattle in Virginia showed clinical signs consistent with a hemoprotozoan infection. We used amplicons specific for the major piroplasm surface protein and small subunit rDNA of piroplasms to test blood samples from the cattle by PCR. Bidirectional Sanger sequencing showed sequences with 100% identity with *T. orientalis* Ikeda genotype 2 sequences. We detected the parasite in 3 unrelated herds and from various animals sampled at 2 time points. Although other benign *T. orientalis* genotypes are endemic to the United States, detection of *T. orientalis* Ikeda genotype might represent a risk for the cattle industry in Virginia.

*Theileria orientalis* is an emerging parasitic pathogen of cattle that was originally identified in the Eastern Hemisphere (*1*). The taxonomy of this group is evolving (*2**–**4*); taxonomic classification relies on sequencing of 2 major genes: the small ribosomal subunit (SSU) and the major piroplasm surface protein (MPSP). Use of the names of *T. orientalis* genotypes Ikeda, Chitose, and Buffeli is embedded in the clinical literature; these designations are used throughout this article. A genotype scheme proposed in 2014 classifies *T. orientalis* into 11 genotypes according to variability in the MPSP gene (*4*); *T. orientalis* genotype Ikeda correlates with genotype 2 (*3**,**5*).

Consistent with other members of the genus, *T. orientalis* is a tickborne hemoprotozoan with a life cycle that affects erythrocytes and leukocytes and contributes to chronic anemia, ill-thrift, and persistent subclinical infections. However, it has not been associated with the lymphoproliferative disease seen with *T. parva* and *T. annulata* (*1*). *Haemaphysalis* spp. ticks are the primary biological vector of *T. orientalis* (*6*) and are believed to be essential for completion of the *T. orientalis* life cycle (*7*), although there is limited evidence suggesting that transmission might occur through flies, lice, or vaccine needles (*1*).

In Asia, New Zealand, and Australia, theileriosis caused by *T. orientalis* is an economically serious disease manifested primarily by loss of revenue from deaths or illness in beef and dairy cattle (*1**,**8**–**10*). Of increasing concern is the Ikeda genotype of *T. orientalis*, which has been implicated as the etiologic agent of infectious bovine anemia (*11**,**12*). In Asia, Australia, and New Zealand, the primary tick vector for the *T. orientalis* Ikeda genotype is *Haemaphysalis longicornis*, which is also known as the Asian longhorned or bush tick.

The Asian longhorned tick was first detected in the United States in August 2017 and has subsequently been detected in New Jersey (*13*), New York, North Carolina, Virginia, West Virginia, Pennsylvania, Maryland, Connecticut, and Arkansas (*14**,**15*). However, examination of archived tick samples has identified *H. longicornis* ticks in the United States since 2010 (*14*). Because of the wide host range of this tick, its bisexual nature, and its ability to reproduce parthenogenetically (*16*), concern is increasing that there are established populations in the mid-Atlantic states.

In September 2017, a beef cattle herd in Virginia was given a diagnosis of anemia and suspected anaplasmosis. Blood samples were negative for *Anaplasma marginale* ticks by PCR, but blood smears showed numerous pleomorphic piroplasms. We report identification and characterization of *T. orientalis* Ikeda genotype 2 from an index farm, in adjacent herds, and 2 other counties in Virginia. Although other genotypes of *T. orientalis* are present in the United States (*17**–**19*), the Ikeda genotype in particular has not been identified in North America and represents an emerging infectious disease with potential for major animal health and economic impacts, especially because a competent vector has been identified in this region and in 2 of the 3 farms described in this report.

## Materials and Methods

### Animals

**Figure 1:**
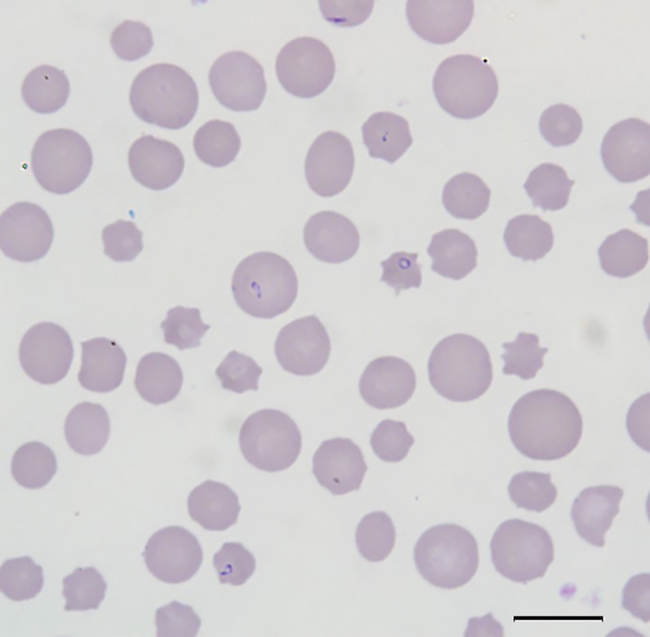
Blood smear of an animal from a farm in Albemarle County, Virginia, USA, that was infected with *Theileria orientalis* Ikeda genotype. There is evidence of a regenerative response to anemia (anisocytosis and polychromasia) and intracellular piroplasms within erythrocytes. Scale bar indicates 10 µm.

All cattle were client-owned animals. In August 2017, seven cattle from a herd in Albemarle County, Virginia, died after showing adverse clinical signs, including weakness and malaise. Affected cattle included bulls, cows, and steers ranging in age from 3 months to 13 years. All animals were born and raised on a farm. In September 2017, an additional cow from the index farm was examined for weakness, icterus, and anemia (packed cell volume [PCV] 12.0%). Blood from this animal was collected and submitted to the Kansas State Veterinary Diagnostic Laboratory (Manhattan, KS, USA). Blood smear analysis showed evidence of a hemoprotozoal infection (Figure 1). Molecular testing of this sample resulted in a diagnosis of infection with *T. orientalis*, which prompted quarantine of the affected farm and further investigation.

A foreign animal disease investigation was instituted during December 2017, and blood was collected from the index cow and 5 additional, randomly sampled cattle from the herd. We collected blood by jugular vein venipuncture from each animal in 10-mL BD Vacutainer plastic red-top tubes containing no anticoagulant and in 10-mL BD Vacutainer plastic purple-top tubes containing EDTA anticoagulant (both from Becton, Dickinson and Company, https://www.bd.com) as part of a routine diagnostic investigation for suspected anaplasmosis or as part of a random sampling effort.

### DNA Extraction and PCR Testing

We extracted DNA from EDTA anticoagulant blood by using the DNeasy Blood and Tissue Kit (QIAGEN, https://www.qiagen.com) according to the nonnucleated blood protocol. We performed the final elution stage by using 50 μL of nuclease-free water and incubating the spin column membrane for 1 min at room temperature. This step was repeated to yield a final elution volume of 100 μL.

We performed amplification for the MPSP by using 10.5 μL of DNA in a 25-μL reaction volume containing primers MPSP forward (5′-CTTTGCCTAGGATACTTCCT-3′) and MPSP reverse (5′-ACGGCAAGTGGTGAGAACT-3′) as described (*11**,**20*). Each amplification had a final reaction primer concentration of 0.4 μmol/L.

We performed amplification for the internal segment of the SSU by using 10 μL of DNA in a 25-μL reaction volume containing primers SSU internal forward (5′-ATTGGAGGGCAAGTCTGGTG-3′) and SSU internal reverse (5′-CTCTCGGCCAAGGATAAACTCG-3′) as described (*11**,**20*) and identical PCR protocols as we described previously. We also used a Biometra TProfessional Thermocycler (AnalytikJena AG, https://www.analytik-jena.com).

We visualized amplicons by electrophoresis on a 1.0% agarose gel containing ethidium bromide and Tris-borate-EDTA buffer and examined amplicons by using UV transillumination. Amplicons with sizes of 700–800 bp were submitted to the Virginia Biocomplexity Institute (Blacksburg, VA, USA) for bidirectional Sanger sequencing.

### Anaplasmosis Testing

Animals sampled as part of a foreign animal disease investigation were tested for *Anaplasma*, *Babesia,* and *Leptospira* spp. at the National Veterinary Services Laboratories (NVSL; Ames, IA, USA). The other animals involved in this study were tested for *Anaplasma marginale* by using a quantitative PCR used and validated by the Virginia Tech Animal Laboratory Services (ViTALS, Blacksburg, VA, USA) diagnostic laboratory (*21*) (Table).

**Table:** Diagnostic testing results for *Theileria orientalis* Ikeda genotype in cattle, Virginia, USA*

Animal ID	Icterus/PCV/parasitemia	*Anaplasma* tick	Origin	Date
I1	Yes /12.0%/6%	Negative (NVSL)	Albemarle (Index farm)	Sep 2017, Dec 2017
I2	No	Negative (NVSL)	Albemarle (Index farm)	Dec 2017, May 2018
I3	No	Negative (NVSL)	Albemarle (Index farm)	Dec 2017, May 2018
I4	No	Negative (ViTALS)	Albemarle (Index farm)	Jul 2018
I5	No	Negative (ViTALS)	Albemarle (Index farm)	Jul 2018
Al1	No	Negative (ViTALS)	Albemarle	Aug 2018
Al2	Yes/10.0%/NA	Negative (ViTALS)	Albemarle	Oct 2018
P1	Yes/14.4%/16.4%	Positive (ViTALS)	Pulaski	Oct 2018
A1	No	Negative (ViTALS)	Augusta	Oct 2018
A2	No	Negative (ViTALS)	Augusta	Oct 2018

*ID, identification; PCV, packed cell volume; NA, not available; NVSL, National Veterinary Services Laboratories; ViTALS, Virginia Tech Animal Laboratory Services.

For the samples tested by ViTALS, we extracted DNA from blood by using the protocol we described previously. DNA samples were diluted 1:10 with nuclease-free water. Amplifications were performed by using AM-For 16S forward primer (5′-TTGGCAAGGCAGCAGCTT-3′) and AM-Rev 16S reverse primer (5′-TTCCGCGAGCATGTGCAT-3′) at a concentration of 0.6 μmol/L each, and AM-Pb probe (5′-6-FAM/TCGGTCTAACATCTCCAGGCTTTCAT/3BHQ_1-3′) at a concentration of 0.2 μmol/L. Reactions were performed in an ABI 7500 Fast thermocycler (Thermo Fisher Scientific, https://www.thermofisher.com) as described (*21*).

### Sequence Analysis

We examined Sanger sequences of SSU and MPSP amplicons for quality and integrity and generated a consensus sequence by using Geneious Prime R11 (Biomatters, https://www.geneious.com). For paired samples of insufficient quality to form a consensus sequence, the highest quality sequence of the 2 sequences was used. We then aligned consensus sequence extractions with sequences of 3 *T. orientalis* Ikeda genotypes (GenBank accession nos. AB581627, AP011946, and D11046) by using Geneious Prime.

### Phylogenetic Analysis

To examine the phylogenetic relationship of the cattle parasite in Virginia with other *Theileria* species, including the 3 genotypes of *T. orientalis*, we constructed a neighbor-joining tree by using a Tamura–Nei genetic distance model with 100 replications. We used the phylogenetic tree for the MPSP gene to best illustrate the relatedness of the samples from cattle in Virginia to the described *T. orientalis* genotypes (*4*). Phylogenetic analysis for the SSU gene included the *T. orientalis* Chitose genotype (GenBank accession no. AB520954), 2 *T. orientalis* Buffeli genotypes (accession nos. AB520955–6), 2 *T. orientalis* Ikeda genotypes (accession nos. AB520957–8), and *T. annulata* (accession no. AY524666) and *T. parva* (accession no. AF013418) as outgroups.

## Results

### Animals

Serologic analysis performed at NVSL for the 6 cattle tested showed negative results for *Anaplasma*, *Babesia*, and *Leptospira* species; all 6 animals were infected with piroplasmid hemoparasites on the basis of blood smear review. Subsequent blood samples were collected during May 2018 from 7 animals within the index herd. Two of these animals previously had intracellular piroplasms; 5 of these animals were sampled randomly. Multiple cattle were infested with ticks, which were morphologically identified as *H. longicornis* by NVSL (*14*). In July 2018, blood samples were collected from 21 cattle representing animals from the index herd and others owned by the same producer on other farms in the county. Before the day of collection, there had been no contact among cattle from different herds. The 21 samples collected represented blood from 20 previously unsampled animals (sampled randomly) and 1 from a calf that had been piroplasm positive during May 2018.

A random sample from a livestock auction from a cow in Albemarle County was submitted during August 2018 (Al1). Four additional samples were submitted from animals in Augusta (A1, A2), Pulaski (P1), and Albemarle (Al2) Counties during October 2018. These animals were unrelated to the cattle at the index farm and had no contact with each other. Of these 5 samples, 3 samples were random samples from a livestock auction (A1, A2, Al1), and 2 samples were submitted for evaluation of clinical disease (P1, Al2). All animals, except P1, were negative for *Anaplasma marginale*. On the basis of a blood smear, P1 had 16% of its erythrocytes parasitized by piroplasms (Table). In addition, this animal had a marked macrocytic, hypochromic, regenerative anemia, hyperbilirubinemia, and icteric plasma consistent with hemolytic anemia (PCV 14.4% [reference range 24.0%–46.0%]; mean corpuscular volume 94.4 fL [reference range 40.0 fL–60.0 fL]; mean corpuscular hemoglobin concentration 27.3 g/dL [reference range 30.0 g/dL–36.0 g/dL]; reticulocytes 194,400/μL [reference range not established]; and total bilirubin 4.1 mg/dL [reference range 0.1 mg/dL–0.6 mg/dL]). Blood smear analysis revealed a 16% parasitemia. P1 died shortly after the blood draw; another animal on the farm had icterus and died 3 days earlier. Al2 had a PCV of 10.0% and showed signs of icterus and lethargy.

### Blood Smears

The blood smear examinations for the cow that became ill during September 2017 showed evidence of a regenerative response to anemia (anisocytosis, polychromasia, basophilic stippling) and numerous oval, racquet, signet ring, and linear piroplasms within erythrocytes (Figure 1). During May 2018, blood smear analysis on 7 additional, randomly sampled animals did not show piroplasms. Two of these samples were from animals that had blood collected and analyzed by NVSL during December 2017; both of these animals were positive for piroplasms in blood smears at that time.

### PCR

Molecular testing of the initial blood sample from the cow that became ill during September 2017 (I1) resulted in a diagnosis of infection with *T. orientalis*. This finding, which, in conjunction with the severity of the clinical signs in this animal and death of previous animals on the premises, led to further investigation of the genotype of the organism and quarantine of the affected farm.

Five of the 6 blood samples taken from cattle during December 2017 were positive by MPSP or SSU assays. Six of 7 cattle from the index herd sampled during May 2018 were positive by MPSP and SSU assays. Of the additional 21 samples collected during July 2018 from the index herd and adjacent properties, only samples from 3 animals were negative by MPSP and SSU assays. Three cattle from Augusta and Pulaski Counties (A1, A2, P1) and 2 cattle from an unrelated herd in Albemarle County (Al1, Al2) were positive by MPSP and SSU assays. A total of 34 sequences were available for analysis, representing 31 unique animals from multiple farms across southwestern Virginia.

Two cattle (I2 and I3) were positive by MPSP and SSU assays during December 2017, and remained positive by these assays during May 2018. One animal was positive by MPSP and SSU assays during May 2018, and remained positive for both assays during July 2018. The presence of positive samples at a sampling interval of 1–5 months suggests a chronic or persistent component of the infection.

### Sequence and Phylogenetic Analysis

**Figure 2:**
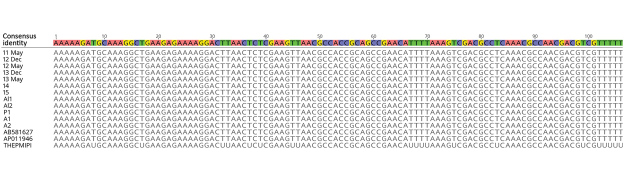
DNA sequences from 10 cattle from a farm in Albemarle County, Virginia, USA, infected with *Theileria orientalis* Ikeda genotype aligned with 3 GenBank sequences of *T. orientalis* genotype 2 for the major piroplasm surface protein. Alignment shows 100% consensus. Samples represent cattle from 6 different herds, and 2 samples were obtained at 2 time points. Pink indicates adenine, yellow indicates guanine, green indicates thymine, and purple indicates cytosine. Nucleotides at the top indicate the consensus sequence. The GenBank sequence THEPMiPI is RNA with uracil substituted for thymine.

All 34 MPSP gene (719 bp) sequences from cattle from multiple farms in all 3 counties in Virginia sampled were identical to each other and to *T. orientalis* Ikeda and genotype 2 sequences from GenBank (Figure 2). MPSP sequences of animals I2 and I3 sampled during December 2017 and May 2018 were identical.

**Figure 3:**
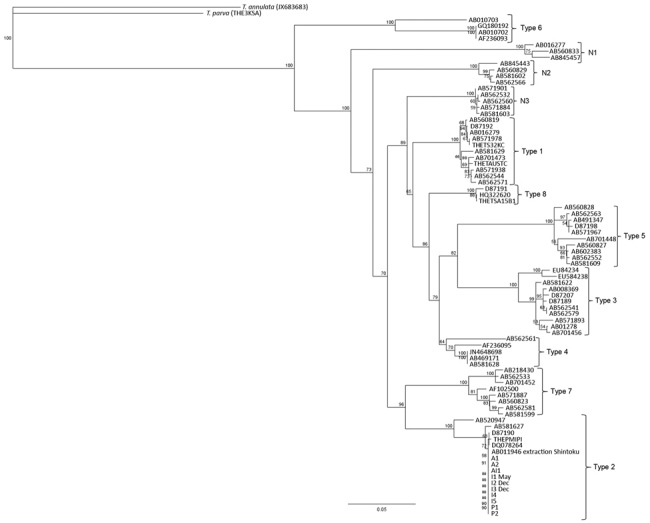
Phylogenetic tree showing major piroplasm surface unit gene sequences for *Theileria* species. The tree uses reference sequences from the major genotypes for *T. orientalis* (*4*). Sequences from infected cattle in Virginia, USA, cluster with genotype 2 sequences. Numbers along branches are bootstrap values. Scale bar indicates nucleotide substitutions per site.

**Figure 4:**
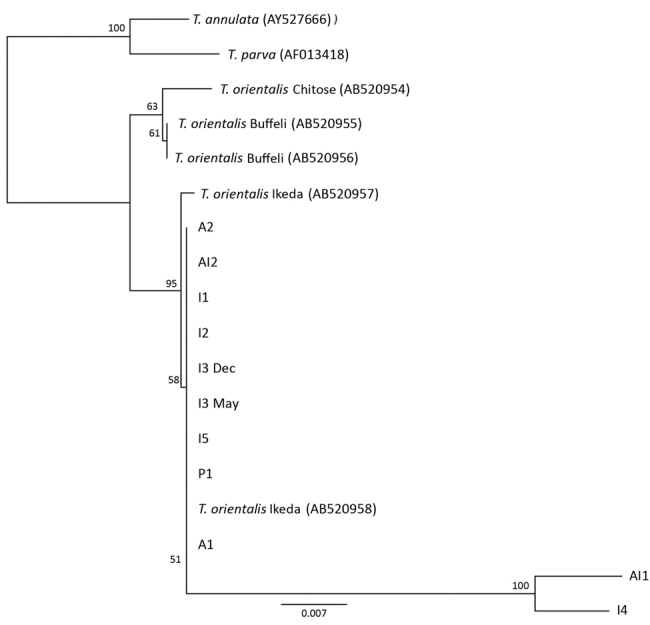
Phylogenetic tree showing small subunit rDNA gene sequences for *Theileria* species. Representative samples from cattle in 6 herds in Virginia, USA, cluster with the reference sequences for the Ikeda genotype. The next most closely related branch is composed of *T. orientalis* Chitose genotype and then *T. orientalis* Buffeli genotype. The outgroup is composed of a single reference sequence each for *T. parva* and *T. annulata*. Small subunit rDNA sequences for I4 and Al1 were of low quality (6.4% and 3.9%, respectively). Numbers along branches are bootstrap values. Scale bar indicates nucleotide substitutions per site.

On the basis of MPSP gene sequence phylogeny reported by Sivakumar et al. (*4*), all clinical samples clustered together, along with *T. orientalis* Ikeda and genotype 2 sequences retrieved from GenBank (Figure 3). The next closest related branch was composed of the cluster of *T. orientalis* genotype 7. Outgroups composed of *T. annulata* and *T. parva* clustered appropriately. On the basis of SSU gene sequence phylogeny (*4*,*22*) all clinical samples clustered together, along with *T. orientalis* Ikeda genotype sequences from GenBank (Figure 4).

## Discussion

Genotypes of *Theileria orientalis* are native to the United States, but are of the Buffeli genotype and are typically nonpathogenic (*17**–**19*). *T. orientalis* Ikeda/genotype 2, a novel, virulent genotype, has not been previously identified in North America. Although the vector of *T. orientalis* among cattle in Virginia is unknown, *H. longicornis* ticks are a major vector in New Zealand, Australia, and Asia (*1*). The recent identification of this tick in the United States and clinical signs of anemia in an *Anaplasma*-negative herd of cattle infested by *H. longicornis* ticks prompted further investigation into the genotype of *T. orientalis* detected in the cattle sampled.

MPSP is an antigenic marker of *Theileria* spp., and is used to genotype the 11 *Theileria* groups (*4**,**5*). The sequences analyzed in this study are phylogenetically consistent with genotype 2, equivalent to the Ikeda genotype. Because the samples examined in this study represent individual cattle from geographically distant herds, there is concern that *T. orientalis* Ikeda/genotype 2 could be widespread in the region. This concern is especially potent given the presence of a known vector, *H. longicornis* ticks, within the region simultaneously (*14*). No ticks from the region have been tested for *T. orientalis*. Thus, further work is needed to better understand transmission of *T. orientalis* Ikeda by *H. longicornis* ticks or other tick species in Virginia.

The presence of *T. orientalis* Ikeda/genotype 2 in Augusta County is of particular concern because this county has the second largest number of cattle in Virginia (*23*). Rockingham County, which produces most of the cattle in Virginia, is adjacent to Augusta and Albemarle Counties. Future studies are needed to determine the presence of *H. longicornis* ticks and *T. orientalis* Ikeda/genotype 2 in this area, but disease transmission to and within this area is of concern to cattle producers in Virginia. In countries in which *T. orientalis* Ikeda is established, the parasite contributes to economic losses through chronically ill animals. During 2010 in Australia, losses caused by *T. orientalis* Ikeda were estimated to be Aus ≈$20 million (*5**,**10*). In New Zealand, the cost of 1 outbreak on a large dairy farm was estimated to be ≈1 million New Zealand dollars (*5*).

In this initial study of cattle in the United States, some of the animals that were positive for *T. orientalis* Ikeda by PCR remained positive 5 months later, suggesting a chronic state, although the animals that had initially exhibited clinical signs were no longer clinically ill during the spring and summer months. In New Zealand, disease caused by *T. orientalis* demonstrates seasonal pathogenicity, and anemia is more pronounced during autumn and winter months (*12*). Whether this trend is the case in Virginia will require future exploration. These cattle were also initially suspected to have anaplasmosis and had additional testing not been conducted, the detection of this pathogenic *Theileria* species might not have occurred. This study highlights the need for more surveillance and appropriate characterization of any parasites detected.

The source and timing of introduction of *T. orientalis* Ikeda/genotype 2 into the United States is unclear. One potential mechanism of introduction is through subclinically infected live cattle imported from disease-endemic regions. Approximately 200 live Wagyu cattle were imported from Japan during 1993–1997 before a ban on exports of Wagyu cattle by Japan in 1997 (*24*). Some of these same cattle were exported to Australia. It is possible that this or other similar live cattle trade introduced this genotype to the United States. Another possibility is transstadial transmission of *H. longicornis* ticks accidentally brought into the United States on other animals in shipments. Other genotypes of *T. orientalis* have been identified in the United States (*14**,**17**,**18*), suggesting that there were previous introductions. Regardless, now that both the tick and the hemoprotozoan are established in this region, *H. longicornis* ticks will probably play a role in continued transmission of the parasite, and *Theileria*-associated bovine infectious anemia will likely continue to occur within the area. A study modeling the most likely suitable habitats of the Asian longhorned tick throughout North America predicts that the tick will thrive along the eastern United States seaboard from Maine to North Carolina, along the western United States seaboard from Washington to northern California, and from northern Louisiana northward into Wisconsin and Ohio (*25*). With such a broad range, we predict *T. orientalis* Ikeda will become established in multiple states throughout the country.
